# A Case of Emetophobia Responding to Selective Serotonin Reuptake Inhibitors: Implications for Treatment

**DOI:** 10.1192/j.eurpsy.2025.1027

**Published:** 2025-08-26

**Authors:** R. A. Moreira, H. J. Gomes, E. Maldonado, I. Machado, J. M. Justo

**Affiliations:** 1 Psiquiatria, Unidade Local de Saúde do Nordeste, Bragança, Portugal

## Abstract

**Introduction:**

Emetophobia, an intense and disproportionate fear of vomiting, is a chronic and debilitating condition characterized by the avoidance of situations or activities that may increase the perceived risk of vomiting. It is often accompanied by an overwhelming fear of losing control, becoming severely ill, or being perceived as repulsive by others. Treating emetophobia is particularly challenging, primarily due to the difficulties in implementing exposure-based interventions effectively. Data on the pharmacological management of emetophobia is limited, with only a few case reports in the literature suggesting potential benefits from selective serotonin reuptake inhibitors (SSRIs).

**Objectives:**

With this case report we aim to describe a case of emetophobia treated with an SSRI.

**Methods:**

Description of a clinical case of a patient with emetophobia observed in a psychiatric outpatient consultation.

**Results:**

We present the case of a 21-year-old male with a one-year history of emetophobia. At the time of consultation, he was unemployed, having recently completed a computer engineering degree. He had no relevant past medical history and was not on any regular medication. The patient was referred for psychiatric evaluation after three years of psychotherapeutic treatment for generalized anxiety disorder. His anxiety symptoms worsened one year prior after an episode of vomiting, which triggered a fear of recurrence. This fear led to restrictive eating, resulting in a 10 kg weight loss over the preceding year (current weight: 63 kg), and significant social avoidance due to the fear of vomiting in public. Physical and neurological examinations revealed no abnormalities. Routine blood tests, urinalysis, and urine drug screening showed normal results. Given the limited efficacy of prior psychotherapeutic interventions, pharmacotherapy with escitalopram 10 mg daily was initiated. After one year of follow-up, the patient reported substantial improvement in anxiety symptoms and avoidance behaviors related to his fear of vomiting. He was able to resume normal eating patterns and gained 5 kg during this period.

**Conclusions:**

This case highlights the potential efficacy of SSRIs, specifically escitalopram, in the treatment of emetophobia. After one year, the patient showed significant improvement, with the return of normal eating patterns and weight gain. While exposure-based interventions remain a cornerstone of treatment for specific phobias, pharmacological options like SSRIs can serve as a valuable adjunct, especially in cases with comorbid anxiety disorders. Further research is needed to better understand SSRIs’ role in managing emetophobia and refine treatment strategies for this complex condition.

**Disclosure of Interest:**

None Declared

